# Association between women’s experience of domestic violence and childhood vaccination in West Africa: Cross-sectional analysis of Demographic and Health Survey data

**DOI:** 10.1371/journal.pone.0293900

**Published:** 2023-11-02

**Authors:** Toluwalogo Daramola, Lisa Szatkowski

**Affiliations:** Academic Unit of Lifespan and Population Health, School of Medicine, University of Nottingham, Nottingham, United Kingdom; Universidade Federal de Minas Gerais, BRAZIL

## Abstract

**Background:**

In 2021, 25 million children worldwide did not receive full basic childhood vaccinations, the highest figure in over a decade. There are large variations between countries in vaccination coverage. Globally, the lifetime prevalence of domestic violence among ever-partnered women is 30%. Exposure to domestic violence affects both maternal and child health. However, there is limited contemporary evidence on whether children born to women who are exposed to domestic violence are any more or less likely to be vaccinated.

**Methods:**

We conducted a cross-sectional study using data from the most recent Demographic and Health Surveys (DHS) from 7 West African countries (Benin, Gambia, Liberia, Mali, Nigeria, Senegal, Sierra Leone). We used multivariable logistic regression to examine the association between women’s lifetime experience of any emotional, physical and/or sexual domestic violence and whether her most-recent born child aged 12–35 months old had received a full complement of basic childhood vaccinations (covering tuberculosis, diphtheria, tetanus, pertussis, polio and measles).

**Results:**

Data from 9,104 mother-child pairs was analysed (range 480 from Senegal to 3,230 from Nigeria). Overall, 47% of children were fully vaccinated (range 31% in Nigeria to 81% in The Gambia). 41% of women reported any experience of domestic violence (range 20% in Senegal to 54% in Sierra Leone). After adjustment for a range of child, maternal, household and partner-level variables, children born to women who reported experience of domestic violence were no more or less likely to be fully vaccinated (adjusted odds ratio = 1.02, 95% confidence interval 0.90–1.17). There was some evidence that the association may vary by country; in Sierra Leone, children born to women who reported experience of domestic violence were significantly less likely to be fully vaccinated (adjusted odds ratio = 0.62, 95%CI 0.44–0.88).

**Conclusions:**

There was no significant association between a woman’s exposure to domestic violence and whether her child was fully vaccinated. Further work is needed to understand the contextual factors which may explain potential variations between countries.

## Introduction

Vaccination has made significant contributions to global health as both an effective and cost-effective way of reducing the incidence of several communicable diseases [1, 2]. In 1974, the World Health Organisation (WHO) established the Expanded Programme on Immunisation (EPI), a global initiative to ensure all children have access to routinely recommended vaccines that protect against morbidity and mortality from six major diseases: diphtheria, pertussis, tetanus, measles, tuberculosis (TB) and polio; today every country in the world has their own vaccination programme to support this aim [3]. Despite this, in 2021 approximately 25 million children globally under the age of 1 year did not receive the full complement of basic childhood vaccines, an increase of 5.9 million compared with 2019, and the highest figure since 2009 [4]. 18 million “zero-dose” children did not receive any vaccines at all, almost all living in low- and middle-income countries primarily in sub-Saharan Africa and South-East Asia [5]. This increase in the number of unvaccinated children is believed to be fuelled by the COVID-19 pandemic, including its effect on already strained healthcare systems and an apparent impact on confidence in vaccination and increase in vaccine hesitancy [4, 6].

**Fig 1:**
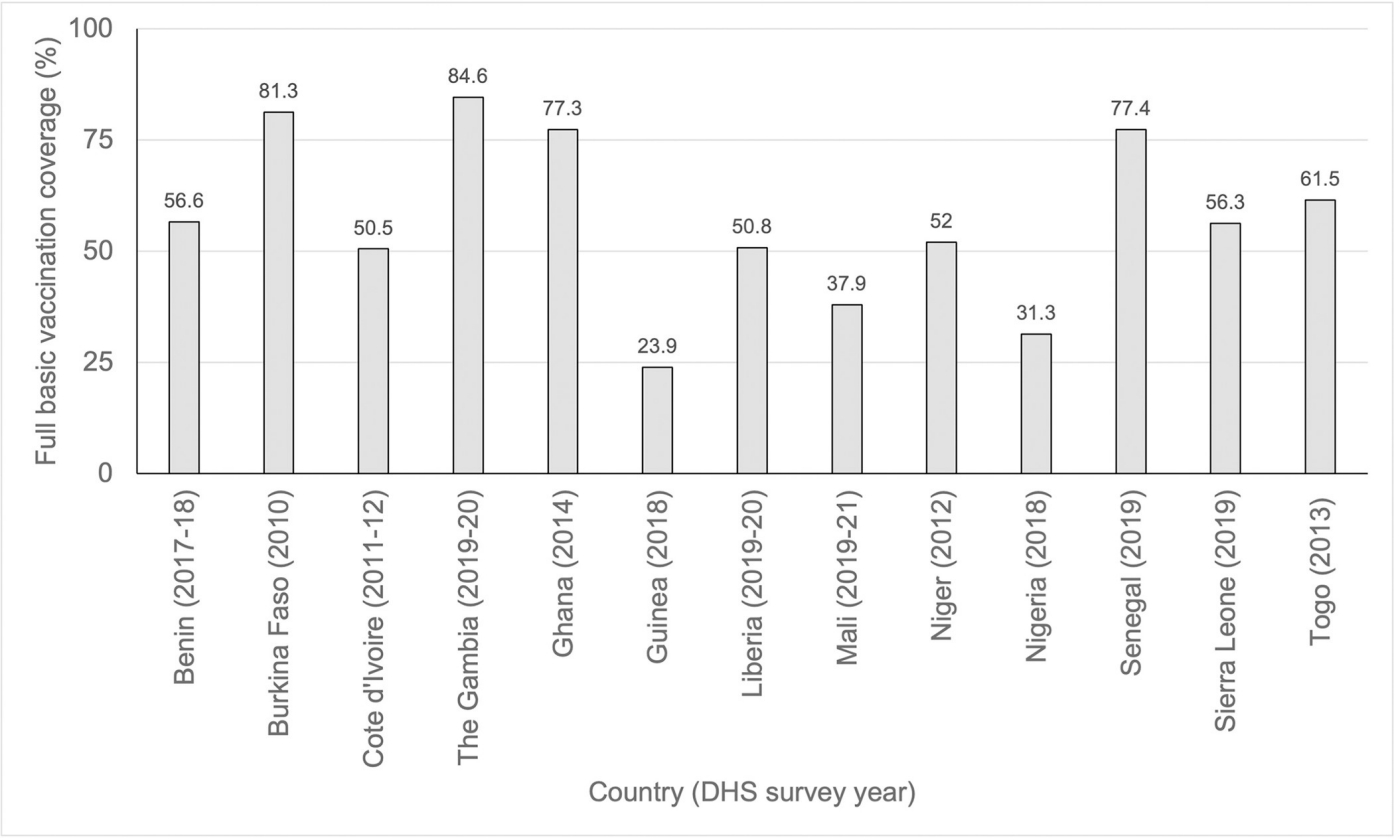
Percentage of children aged 12–23 months in ECOWAS countries who have received all 8 basic childhood vaccinations (based on data from Demographic and Health Surveys [8], no data available for Cape Verde and Guinea-Bissau).

The Economic Community of West African States (ECOWAS) consists of 15 culturally diverse countries with shared colonial histories and geopolitical and economic alliances [7]. The most recent Demographic and Health Survey (DHS) data for these countries show marked differences in basic childhood vaccination coverage, ranging from 23.9% in Guinea (2018) to 84.6% in the Gambia (2019–20) (Fig 1) [8].

In Sub-Saharan Africa, challenges to achieving complete childhood vaccination coverage can be grouped into three broad categories [9]: 1) barriers which prevent parents/caregivers from accessing and utilising vaccination services for their children; 2) logistical and infrastructure barriers related to healthcare systems, which limit vaccine delivery and the effectiveness of vaccine services; and 3) factors which hinder the ability of healthcare providers and workers to correctly use vaccines. Women’s exposure to domestic violence (or intimate partner violence, IPV, the terms are often used interchangeably) is one potential challenge within the first of these categories.

Domestic violence can be defined as “behaviour within an intimate relationship that causes physical, sexual or psychological harm, including acts of physical aggression, sexual coercion, psychological abuse and controlling behaviours” [10]. Domestic violence is usually perpetrated by men against women [10] and is viewed as a major public health issue due to its negative impact on mental and physical health [10–13]. Global estimates (based on data from 2000–2018) suggest that the global lifetime prevalence of physical and/or sexual violence from a current or former partner among ever-partnered women aged 15+ is 26% (95% uncertainty interval (UI) 22–30) [14]. Of all low- and middle-income countries, those in the African region (alongside those in South-East Asia) have the highest lifetime prevalence of domestic violence (prevalence 32%, 95% UI 28–37) [14]. These figures are likely to be underestimates of the true prevalence of domestic violence, as some women will not disclose their experiences.

Domestic violence has a negative effect on maternal health. Women who have experienced domestic violence experience more health issues and consequently incur higher healthcare costs over their lifetime [15], compounded by loss of earnings due to reduced productivity, absenteeism and the impact of long term disabilities. Exposure to domestic violence is strongly linked with risky health behaviours such as alcohol and drug misuse [12, 15, 16].

Exposure to domestic violence may also have a negative impact on the health of children, through both direct and indirect mechanisms [11, 17]. Women who have experienced domestic violence are less likely to receive adequate antenatal care during pregnancy [18, 19] and less likely to deliver in a healthcare facility [19] compared to women who have not experienced domestic violence, compromising the health of both mother and child. Previous studies have reported reduced rates of vaccination amongst the children of women who have experienced domestic violence, though the association may be dependent on the country [17, 20–22]. There is a paucity of contemporary data on the association between experience of domestic violence and childhood vaccination in West Africa, despite the need to tackle the persistently low vaccination coverage. Understanding the contemporary situation may help to identify factors amenable to intervention to improve childhood vaccination coverage. This study uses recent Demographic and Health Survey (DHS) data from several West African countries to fill this evidence gap.

## Methods

### Data source and study population

We used data from the DHS Program of large-scale, nationally representative, standardised household surveys [23]. We restricted analysis to the seven West African countries where a survey was carried out within the past five years: Benin (2017–18), Gambia (2019–20), Liberia (2019–20), Mali (2018), Nigeria (2018), Senegal (2019) and Sierra Leone (2019). The DHS employs a multistage sampling design with a target population of all women aged 15–49 and all children under five years of age living in residential households. Women in households selected for interview are asked about the vaccinations received by their children born in the last 3 years. We identified all children who were alive and aged 12–35 months at the time of the DHS Survey, about whom data was collected from their mother. Basic childhood vaccinations are included within the vaccination schedule for the first year of life, and so infants under one year of age, who may not yet be of the age to receive some vaccinations, were excluded. In households with more than one eligible child we selected the child born most recently for analysis. Children were excluded from the analysis if their mother was not selected and interviewed for the domestic violence survey module.

### Definition of outcome variable—Full basic childhood vaccination

Information on childhood vaccinations was collected in the DHS in two ways: 1) inspection of vaccination record cards by the interviewer; 2) maternal recall where vaccination cards were unavailable. We created a binary variable to indicate whether each child had received a full course of basic childhood vaccinations, defined according to WHO recommendations(3) as: one dose of BCG (Bacillus Calmette-Guérin, tuberculosis vaccine); three doses of diphtheria-tetanus-pertussis containing vaccine (DTP); three doses of polio vaccine (excluding the oral polio vaccine given at birth); one dose of measles-containing vaccine. Where there was missing data for one or more of these vaccines the child was deemed not to be fully vaccinated.

### Definition of exposure variable—Exposure to domestic violence

A binary variable was created to identify whether or not women self-reported having ever experienced any domestic violence across one or more of three domains: emotional violence; physical violence; sexual violence. Where there was missing data for one or more of these domains, and no reported experience of violence in the domains where information was available, the woman’s overall experience of domestic violence was categorised as unknown. Full details of the questions used in the DHS to ascertain women’s experience of domestic violence are given in S1 File.

### Statistical analysis

All data management and analysis was carried out using Stata 17 (Stata Corp., College Station, TX). The DHS sampling design and survey weights were accounted for in the analysis.

The characteristics of the study population were first described, using percentages for categorical variables, mean (standard deviation) for normally distributed continuous variables, and median (interquartile range) for non-normally distributed continuous variables. All data presented account for the survey design, including weighting. Data are presented for both the seven countries combined as well as by individual country. The prevalence of experience of domestic violence, overall and within each individual domain, was also calculated.

Multivariable logistic regression was used to estimate odds ratios with 95% confidence intervals (95% CIs) to quantify the association between a woman’s reported experience of any domestic violence and whether her children were fully vaccinated. Models were adjusted sequentially for characteristics of the child (e.g., sex and birth order), mother (e.g., age, education, employment, marital status), household (urban or rural, and wealth quintile) and, where applicable, the mother’s current partner (age, education, employment, alcohol use). Full details on the coding of these confounding variables is given in S1 File. An interaction term was included in the model to assess whether the impact of experience of domestic violence varied by country and data are presented both for the countries combined as well as by individual country. We defined a threshold for significance (alpha) of 0.05.

### Ethical approval

The DHS Program received ethical approval for the original survey data collection from relevant country ethical review boards. No further ethical approval was required for this study, but permission to use the data was received from the DHS archivist (date of approval 29 March 2021).

## Results

Data were collected on 87,543 children as part of the DHS surveys in the seven countries included in the analysis. Exclusions, for the reasons outlined above, are shown in Table 1. In total, data on 9,104 mother-child pairs was included in the final analysis, ranging from 480 from Senegal to 3,230 from Nigeria.

**Table 1:** Counts of numbers of children surveyed in DHS and included in study.

	All countries	Benin	The Gambia	Liberia	Mali	Nigeria	Senegal	Sierra Leone
**Total number of children aged 0–59 months on whom data collected**	**87,543**	**13,589**	**8,362**	**5,704**	**9,940**	**33,924**	**6,125**	**9,899**
**Total number of children excluded** *	**78,439**	**12,027**	**7,775**	**4,933**	**8,732**	**30,694**	**5,645**	**8,633**
*Mother not interviewed for DV module*	*57*,*933*	*8*,*386*	*6*,*452*	*3*,*256*	*5*,*954*	*23*,*330*	*4*,*686*	*5*,*869*
*Child not alive at time of survey*	*6*,*770*	*938*	*435*	*459*	*665*	*3*,*211*	*226*	*836*
*Child not aged 12–35 months at time of survey*	*53*,*426*	*8*,*368*	*5*,*114*	*3*,*473*	*6*,*033*	*20*,*841*	*3*,*632*	*5*,*965*
*Child not the youngest eligible child in households with 2+ eligible children*	*60*,*688*	*9*,*507*	*5*,*768*	*3*,*880*	*6*,*919*	*23*,*885*	*4*,*077*	*6*,*652*
**Total number of mother-child pairs included in study**	**9,104**	**1,562**	**587**	**771**	**1,208**	**3,230**	**480**	**1,266**
**Number of women with husband/partner**	**8,370**	**1478**	**552**	**566**	**1,160**	**3,054**	**459**	**1,101**

*Numbers of exclusions for individual reasons exceed the total as some children were excluded on the basis of more than one reason

Abbreviations: DV, domestic violence

Table 2 describes the socio-demographic characteristics of the study population. There were substantial differences between countries, but overall the majority of children were male, born in a government/public health facility, the mother had received fewer than the recommended eight antenatal visits, and had not received a postnatal check within two months of birth. Most households were rural and from the poorest wealth quintile. Women were predominantly married, had a median age of 29 and entered into their first union before the age of 20. The majority had no education, were not currently working, did not have full participation in household decision making and most had experienced controlling behaviour from their partner. Partners were, on average, older than the women (median age 37), and whilst almost half had no education, just over a quarter reported secondary education and almost all were currently working.

**Table 2:** Characteristics of study population–children, mothers, households and partners (all figures weighted percentages unless stated otherwise).

	All countries	Benin	The Gambia	Liberia	Mali	Nigeria	Senegal	Sierra Leone
**CHARACTERISTICS OF CHILDREN**								
**n**	9,104	1,562	587	771	1,208	3,230	480	1,266
**Age, months, mean (SD)**	22 (7)	23 (7)	22 (7)	23 (7)	21 (6)	22 (7)	22 (8)	22 (7)
**Male**	51.4	48.8	53.6	50.2	50.7	54.5	47.7	48.2
**Place of birth**								
Home	30.6	12.0	13.7	18.4	28.9	53.9	14.6	13.4
Government/ public health facility	57.5	71.2	77.9	69.0	65.9	28.5	82.2	83.9
Private/ other health facility/ unknown	12.0	16.8	8.4	12.6	5.2	17.5	3.2	2.7
**Number of antenatal visits**								
<8	81.2	88.4	96.1	70.3	95.5	75.5	99.0	64.8
8+	15.6	8.7	2.9	28.6	1.9	23.0	0.2	23.3
Missing	3.2	2.9	0.9	1.1	2.6	1.6	0.8	11.9
**Received postnatal baby check within 2 months**								
No	68.1	79.0	45.2	74.3	74.3	77.2	12.1	50.0
Yes	31.4	20.7	54.7	23.1	25.3	22.4	87.9	49.8
Don’t know	0.5	0.4	0.1	2.7	0.5	0.4	0.0	0.3
**CHARACTERISTICS OF MOTHERS**								
**n**	9,104	1,562	587	771	1,208	3,230	480	1,266
**Age, years, median (IQR)**	29 (25–35)	29 (25–34)	29 (25–34)	27(22–34)	28 (24–34)	30 (25–35)	30 (24–35)	28 (24–35)
**Age at first union**								
<15	19.1	16.7	9.0	22.7	19.4	18.6	11.5	28.5
15–19	65.2	69.9	66.0	73.5	70.5	61.6	45.6	65.0
20–24	12.1	12.7	20.8	3.2	7.9	14.5	31.2	4.2
25–29	1.6	0.5	3.4	0.2	0.3	2.5	7.4	0.1
≥ 30	0.2	0.1	0.6	0.0	0.0	0.3	1.7	0.0
Missing	1.7	0.1	0.3	0.5	2.0	2.6	2.6	2.2
**Number of children ever born, median (IQR)**	3 (2–5)	3 (2–5)	3 (2–5)	3 (2–5)	4 (2–6)	3 (2–6)	3 (2–5)	3 (2–5)
**Religion**								
Catholic or other Christian	33.8	50.9	2.4	84.7	1.7	42.3	2.7	21.1
Islam	62.2	31.8	97.6	13.4	93.6	57.3	97.3	78.9
Traditionalist/ other/ none	4.0	17.3	0.0	1.9	4.8	0.5	0.1	0.0
**Highest level of education**								
No education	51.9	64.0	40.8	39.4	70.7	41.7	59.7	52.5
Primary education	16.8	18.4	19.8	24.5	13.5	14.8	23.4	16.0
Secondary education	26.7	16.5	33.8	34.0	14.0	34.8	14.8	28.6
Higher education	4.6	1.2	5.6	2.2	1.8	8.8	2.1	2.9
**Currently working**	70.3	82.3	55.0	67.1	60.3	70.8	46.6	81.3
**Full participation in decision making?**								
No	62.8	61.4	68.2	16.4	84.8	64.0	88.2	51.9
Yes	27.9	31.7	21.9	48.0	11.1	31.0	7.6	32.0
Missing	9.3	6.9	9.9	35.6	4.1	5.1	4.2	16.2
**Control over own earnings?**								
Yes	45.0	61.1	42.5	27.2	34.5	56.0	37.4	20.4
No	5.8	5.5	5.8	2.8	6.2	5.3	2.1	10.1
Missing	49.3	33.4	51.7	70.0	59.3	38.7	60.5	69.5
**Is any justification for wife-beating acceptable?**								
No	55.5	65.7	44.5	54.8	17.2	70.7	56.7	49.4
Yes	44.2	33.5	55.5	44.9	82.6	29.2	43.3	50.3
Missing	0.3	0.8	0.0	0.3	0.3	0.2	0.0	0.3
**Ever experienced any controlling behaviour from partner?**								
No	33.9	33.1	37.8	15.5	33.3	38.9	73.1	17.2
Yes	60.0	63.6	55.1	58.2	63.3	58.8	25.6	70.3
Missing	6.1	3.3	7.1	26.3	3.4	2.3	1.3	12.5
**Marital status**								
Never in union	5.8	2.7	6.4	26.0	3.2	2.2	1.3	12.3
Married	83.0	74.0	90.1	33.7	95.7	91.6	95.8	77.8
Living with partner	7.7	19.1	0.0	30.7	0.2	3.3	0.0	6.1
Widowed	1.0	1.5	1.4	1.6	0.3	1.0	0.3	1.0
Divorced	0.8	0.6	1.7	0.3	0.4	1.1	2.0	0.2
No longer living together/ separated	1.7	2.2	0.3	7.8	0.2	0.8	0.6	2.7
**Partner has 1 or more other wives**								
No	63.3	58.6	62.1	56.4	59.1	69.9	69.8	58.1
Yes	27.0	33.2	28.0	6.8	36.7	24.9	26.0	25.6
Missing	9.7	8.3	9.9	36.8	4.2	5.2	4.2	16.4
**CHARACTERISTICS OF HOUSEHOLDS**								
**n**	9,104	1,562	587	771	1,208	3,230	480	1,266
Urban	40.7	39.3	66.3	52.9	21.6	44.2	42.9	34.8
**Wealth quintile**								
Poorest	20.8	20.1	23.2	23.6	18.5	19.8	24.1	23.4
Poor	20.2	19.0	22.6	19.8	21.9	19.8	15.3	21.5
Middle	21.2	22.8	18.7	21.0	21.5	21.0	18.0	22.0
Richer	20.2	20.2	19.7	21.8	19.9	20.9	21.3	18.0
Richest	17.5	18.0	15.9	13.8	18.2	18.6	21.4	15.1
**CHARACTERISTICS OF PARTNERS**								
**n**	8,370	1478	552	566	1,160	3,054	459	1,101
**Age, years, median (IQR)**	37 (32–45)	35 (30–40)	40 (35–48)	36 (30–42)	38 (32–45)	38 (32–44)	40 (34–48)	38 (32–45)
**Highest level of education**								
No education	46.7	52.8	47.6	28.8	72.3	30.6	66.2	54.2
Primary education	13.1	20.3	8.3	15.6	8.5	14.3	7.1	8.7
Secondary education	26.9	17.3	25.5	40.4	11.7	38.6	11.8	25.5
Higher education	9.5	5.8	6.2	9.3	3.5	15.3	5.5	8.0
Missing	3.7	3.8	12.4	6.0	4.0	1.2	9.3	3.6
**Currently working**								
No	8.1	4.6	10.9	11.1	12.9	7.2	4.7	8.7
Yes	91.5	95.2	87.9	88.6	86.0	92.6	95.1	91.2
Missing	0.4	0.2	1.2	0.4	1.1	0.2	0.3	0.1
**Drinks alcohol**								
No	78.1	70.8	92.8	47.9	92.9	77.9	97.8	75.0
Yes	16.1	26.6	0.8	26.1	3.9	19.9	0.9	12.7
Missing	5.8	2.7	6.4	26.0	3.2	2.2	1.3	12.3

The overall prevalence of experience of any domestic violence was 41.1% though again there were substantial difference between countries (ranging from 20.0% in Senegal to 54.4% in Sierra Leone). Experience of emotional violence was most common, closely followed by physical violence; experience of sexual violence was less common (Table 3).

**Table 3:** Prevalence of experience of domestic violence and full basic childhood vaccination (all figures weighted percentages).

	All countries	Benin	The Gambia	Liberia	Mali	Nigeria	Senegal	Sierra Leone
**Unweighted sample size**	9,104	1,562	587	771	1,208	3,230	480	1,266
**Mother ever experienced any domestic violence**								
No	53.0	55.6	53.8	32.1	46.9	62.7	78.6	33.3
Yes	41.1	41.7	39.8	41.9	49.8	35.2	20.0	54.4
Missing	5.8	2.7	6.4	26.0	3.2	2.2	1.3	12.3
**Mother ever experienced any emotional violence**								
No	61.6	60.9	71.4	46.1	60.0	66.9	88.6	45.3
Yes	32.5	36.4	22.2	27.9	36.8	30.9	10.1	42.4
Missing	5.8	2.7	6.4	26.0	3.2	2.2	1.3	12.3
**Mother ever *experienced physical violence (severe and/or less severe)***								
No	67.9	79.6	62.2	38.1	58.4	79.8	84.5	45.1
Yes	26.3	17.8	31.3	35.9	38.4	18.0	14.2	42.7
Missing	5.8	2.7	6.4	26.0	3.2	2.2	1.3	12.3
**Mother ever experienced any sexual violence**								
No	86.7	88.1	86.6	67.7	84.9	92.2	94.7	80.5
Yes	7.5	9.3	7.0	6.3	11.9	5.6	3.9	7.2
Missing	5.8	2.7	6.4	26.0	3.2	2.2	1.3	12.3
**Child received full basic childhood vaccination**								
No	52.6	41.8	19.3	51.7	57.1	68.6	22.5	44.7
Yes	47.4	58.2	80.7	48.3	42.9	31.4	77.5	55.3

Overall, 47.4% of children had received a full course of basic childhood vaccinations, ranging from 31.4% in Nigeria to 80.7% in The Gambia (Table 3).

**Fig 2:**
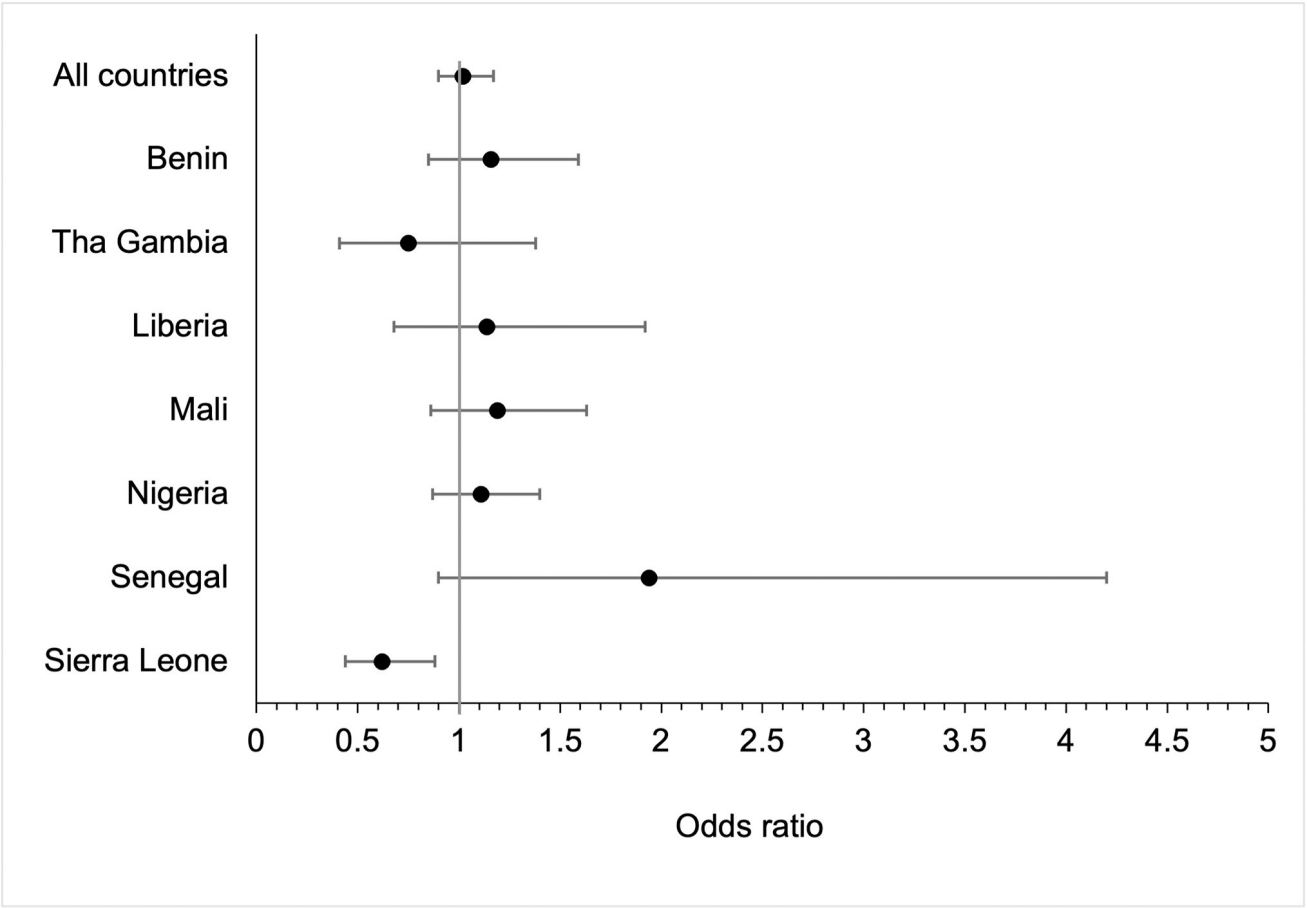
Adjusted odds ratios and 95% confidence intervals showing the association between experience of domestic violence and full basic childhood vaccination (adjusted for child, maternal, household and partner characteristics).

Table 4 and Fig 2 show odds ratios (with 95% CIs) to describe the association between women’s experience of domestic violence and full basic childhood vaccination. Overall, both before and after adjustment, children born to mothers who had experienced domestic violence were no more or less likely to be fully vaccinated than children born to mothers who had not experienced domestic violence (odds ratio from fully adjusted model: 1.02, 95% CI 0.90–1.17). There was no overall evidence of effect modification by country (p-value for interaction 0.083). However, children in Sierra Leone born to mothers who had experienced domestic violence were less likely to be fully vaccinated than children born to mothers who had not experienced domestic violence (odds ratio from fully adjusted model: 0.62, 95% CI 0.44–0.88).

**Table 4:** Adjusted odds ratios and 95% confidence intervals showing the association between experience of domestic violence and full basic childhood vaccination.

	Adjusted odds ratio (95% confidence interval) for association between experience of domestic violence and full basic childhood vaccination				
	Model I	Model II	Model III	Model IV	Model V
**Covariates included in model**	country	country; child characteristics	country; child characteristics; maternal characteristics	country; child characteristics; maternal characteristics; household characteristics	country; child characteristics; maternal characteristics; household characteristics; partner characteristics
**All countries**	1.03 (0.92–1.15)	1.06 (0.94–1.19)	1.03 (0.91–1.18)	1.04 (0.92–1.19)	1.02 (0.90–1.17)
**p-value for interaction**	0.158	0.237	0.113	0.116	0.083
**Benin**	1.15 (0.90–1.46)	1.23 (0.94–1.62)	1.21 (0.90–1.61)	1.22 (0.91–1.64)	1.16 (0.85–1.59)
**The Gambia**	0.95 (0.51–1.76)	0.87 (0.46–1.65)	0.73 (0.39–1.34)	0.72 (0.38–1.36)	0.75 (0.41–1.38)
**Liberia**	0.88 (0.58–1.34)	0.91 (0.56–1.46)	1.05 (0.63–1.74)	1.10 (0.65–1.84)	1.14 (0.68–1.92)
**Mali**	1.29 (0.98–1.69)	1.35 (1.01–1.79)*	1.17 (0.86–1.60)	1.16 (0.85–1.59)	1.19 (0.86–1.63)
**Nigeria**	1.04 (0.86–1.25)	1.04 (0.85–1.28)	1.04 (0.83–1.31)	1.09 (0.86–1.38)	1.11 (0.87–1.40)
**Senegal**	1.01 (0.49–2.08)	1.26 (0.64–2.50)	1.74 (0.81–3.76)	1.77 (0.83–3.80)	1.94 (0.90–4.20)
**Sierra Leone**	0.71 (0.52–0.97)*	0.75 (0.54–1.04)	0.61 (0.43–0.86)*	0.61 (0.43–0.87)*	0.62 (0.44–0.88)*

*p<0.05

## Discussion

Our results show the high prevalence of both exposure to domestic violence and low vaccination coverage in West Africa, both of which have several negative consequences in and of themselves. Overall, there was no significant association between maternal experience of domestic violence and whether her child had received a full course of basic childhood vaccinations. However, the data point to potential differences between countries. In Sierra Leone, children born to mothers who have ever experienced domestic violence were less likely to be fully vaccinated.

Our results align with those of earlier work showing that in the majority of countries there was no statistically significant association between a mother’s exposure to domestic violence and the odds of their child receiving a full course of basic vaccinations [21]. However, in this previous study, in the Dominican Republic and Kenya children born to women with a history of exposure to domestic violence were 48% and 32% less likely, respectively, to be fully vaccinated compared to children born to women with no experience of domestic violence [21]. The magnitude of effect seen in Sierra Leone in our study, a 38% reduction in the odds of full vaccination, is similar.

Experience of domestic violence may conceivably reduce the likelihood of childhood vaccination by disempowering women and reducing their agency and ability to make decisions and access appropriate healthcare to meet their and their children’s needs [24]. Exposure to domestic violence may also increase the risk of poor mental health in mothers [25] and negatively impact women’s attachment and parenting capabilities, thereby also reducing the likelihood of their children being vaccinated [24].

Our findings support the suggestion that the association between experience of domestic violence and basic childhood vaccination may be dependent on context; in some geographical locations there may be other factors that have a stronger influence on whether a child is vaccinated than their mother’s experience of domestic violence. The magnitude of differences in vaccination behaviour between women exposed and not exposed to domestic violence may also reflect the overall completeness of vaccination coverage in any given country. Nigeria, for example, has the lowest overall prevalence of complete basic childhood vaccination. Here, structural barriers may prevent women from vaccinating their children regardless of their experience of domestic violence or willingness and desire to vaccinate. Decentralisation, poor governance and ineffective management of the healthcare system may act as barriers to vaccination for all women and children [26, 27].

In Sierra Leone, overall vaccination coverage is far from complete, despite the introduction in 2010 of an initiative removing financial barriers to accessing healthcare for all pregnant women, lactating mothers and children under the age of 5 [28]. The 2014–2015 Ebola outbreak led to an increase in the number of teenage pregnancies due to school closures and increased exposure of young girls to violence and sexual exploitation from men [29]. 42% of girls aged 15–17 years became pregnant for the first time during this outbreak [30]. Young maternal age associated with a decreased likelihood of childhood vaccination [31] and women’s self-reported exposure to domestic violence also varies by age [14]. Whilst we have adjusted for maternal age in our analysis, the relatively small DHS samples preclude adequately-powered subgroup analyses; we have not, therefore, been able to explore whether the inverse association between exposure to domestic violence and childhood vaccination is concentrated amongst younger women who became mothers during the Ebola outbreak.

This study is the most comprehensive recent exploration of the association between maternal experience of domestic violence and childhood vaccination in West Africa. The standardisation and pre-testing of the DHS survey instruments enables compilation and comparison of results from countries, and vaccination data were well recorded (only 67 infants were missing data for one or more of the 8 basic vaccinations). The wide range of data collected enables adjustment for a range of sociodemographic and contextual factors which may confound the association between the exposure and the outcome. However, we were not able to adjust for many additional potential confounders where information was not gathered by the DHS questionnaires. For example, in Mali, parents commonly cite insufficient money to pay for vaccination cards and transport, as well as a lack of information about the vaccinations, waiting times at vaccination centres, and the unfriendliness of vaccination staff, as reasons for their children not being vaccinated [32]. Similar barriers are perceived by women in Nigeria, such as the need to travel long distances to vaccination clinics [33], clinic understaffing leading to long waiting times [34, 35] and disrepair of vaccination clinics and facilities [36]. On the other hand, in The Gambia the provision of vaccination services via ‘trekking’ (outreach) clinics may serve to increase vaccination rates [37]. Data on variables such as these are not available within the DHS. Additionally, the relatively small absolute sample size, particularly in some countries, limits the ability to adjust for multiple confounders and may limit the power to detect associations as statistically significant. In The Gambia, the estimate for the odds ratio also points towards a detrimental effect of exposure to domestic violence on childhood vaccination, though the sample size was small and confidence interval wide.

The cross-sectional design of the DHS limits the ability to establish temporal relationships between maternal experience of domestic violence and childhood vaccination; women are asked whether they have *ever* experienced domestic violence, and so some women’s experience may be more recent than vaccination of their children. When administering the DHS survey, fieldworkers are trained to terminate the interview if a woman’s privacy cannot be ensured or maintained. Though vital to ensure respondents’ safety, this leads to the systematic omission of women from households in which privacy is not possible, such as women living in crowded households. Household crowding is an indicator of low socioeconomic status and stressful living conditions [38], factors which are independently associated with an increased likelihood of experiencing domestic violence. Therefore, omitting women for whom privacy could not be ensured may lead to an underestimation of the prevalence of domestic violence. In addition, information on vaccination is collected partly through maternal recall; recall and/or response bias may affect the validity of this data [39].

## Conclusions

Whilst our study shows no significant association between women’s experience of domestic violence and childhood vaccination, this may vary according to local context. Qualitative work with women may be helpful to unpick the potential association further and identify factors amenable to intervention to help improve childhood vaccination coverage in West Africa.

## Supporting information

S1 File Derivation of variables. (DOCX)
