# Supplementary material for: Association between women’s experience of domestic violence and childhood vaccination in West Africa: Cross-sectional analysis of Demographic and Health Survey data
Source: PLoS One. 2023 Nov 2;18(11):e0293900. doi: 10.1371/journal.pone.0293900 (PMC10621962; doi:10.1371/journal.pone.0293900)
Supplement: S1 File — (DOCX) [file pone.0293900.s001.docx]

**Association between women’s experience of domestic violence and childhood vaccination in West Africa: cross-sectional analysis of DHS data**

**Supporting information file S1: Derivation of variables**

**Table S1: Derivation of experience of domestic violence**

| Variable | DHS question(s) |
| --- | --- |
| Emotional violence | Did your (last) (husband/partner) ever:   1. Say or do something to humiliate you in front of others? 2. Threaten to hurt or harm you or someone you care about? 3. Insult you or make you feel bad about yourself?   Women who answered “*yes*” to any of these questions were considered to have ever experienced any emotional violence. |
| Physical violence | Did your (last) (husband/partner) ever do any of the following things to you:   1. Push you, shake you, or throw something at you? 2. Slap you? 3. Twist your arm or pull your hair? 4. Punch you with his fist or with something that could hurt you? 5. Kick you, drag you, or beat you up? 6. Try to choke you or burn you on purpose? 7. Threaten or attack you with a knife, gun or other weapon?   Women who answered “*yes*” to questions a-d were considered to have ever experienced any less severe violence, and those who answered “*yes*” to questions e-g were considered to have ever experienced any severe violence. |
| Sexual violence | Did your (last) (husband/partner) ever:   1. Physically force you to have sexual intercourse with him when you did not want to? 2. Physically force you to perform any other sexual acts that you did not want to? 3. Force you with threats or in any other way to perform sexual acts you did not want to?   Women who answered “*yes*” to any of these questions were considered to have ever experienced any sexual violence. |

**Table S2: Derivation of other confounding variables**

| **Variable** | **DHS question(s)** | **Categorisation for analysis** |
| --- | --- | --- |
| **CHARACTERISTICS OF CHILDREN** | | |
| Age | Is (CHILD’S NAME) still alive? How old was (CHILD’S NAME) at (his/her) last birthday? | 1. 12-23 months 2. 24-35 months |
| Sex | Is (CHILD’S NAME) a boy or a girl? | 1. Male 2. Female |
| Number of antenatal visits | How many times did you receive antenatal care during this pregnancy? | Reported as continuous data, then categorised according to WHO recommendation that women should receive at least 8 antenatal contacts during pregnancy:   1. <8 2. 8+ 3. Missing |
| Place of birth | Where did you give birth to (CHILD’S NAME)? | 1. Home 2. Government/ public health facility 3. Private/ other health facility/ unknown |
| Received postnatal baby check within 2 months | Did any health care provider or a traditional birth attendant check on (CHILD’S NAME)’s health in the two months after you left (FACILITY IN WHICH WOMAN GAVE BIRTH)? | 1. No 2. Yes 3. Don’t know |
| **CHARACTERISTICS OF MOTHERS** | | |
| Age | How old were you at your last birthday? | 1. 15-19 2. 20-24 3. 25-29 4. 30-34 5. 35-39 6. 40+ |
| Age at first union | How old were you when you had sexual intercourse for the very first time? | 1. <15 2. 15-19 3. 20-24 4. 25-29 5. ≥ 30 6. Missing |
| Total number of children ever born | Have you ever given birth? Followed by multiple questions to identify number of sons and daughters alive and living with them, alive and not living with them, and born alive but who later died. Final question: Just to make sure that I have this right: you have had in TOTAL births during your life. Is that correct? | Reported as continuous data, then split into 3 groups at 25^th^ and 75^th^ quartiles:   1. 1 2. 2-5 3. 6+ |
| Religion | What is your religion? | Free text response grouped into major categories:   1. Catholic or other Christian 2. Islam 3. Traditionalist/ other/ none |
| Highest level of education | Have you ever attended school? What is the highest level of school you attended:  primary, secondary, or higher? | 1. No education 2. Primary education 3. Secondary education 4. Higher education |
| Currently working | Aside from your own housework, have you done any work in the last 7 days? | 1. No 2. Yes 3. Missing |
| Do you have full participation in decision making? | Who usually makes decisions about the following thigs: you, your (husband/partner), you and your (husband/partner) jointly, or someone else?   1. health care for yourself 2. making major household purchases 3. visits to your family or relatives | Mother’s full participation deemed as a positive response in all 3 areas:   1. No 2. Yes 3. Missing |
| Do you have control over own earnings? | Who usually decides how the money you earn will be used: you, your (husband/partner), or you and your (husband/partner) jointly? | 1. No (husband/partner alone) 2. Yes (respondent alone or respondent and husband/partner jointly) 3. Missing |
| View on whether any justification for wife-beating is acceptable? | In your opinion, is a husband justified in hitting or beating his wife in the following situations:   1. if she goes out without telling him? 2. if she neglects the children? 3. if she argues with him? 4. if she refuses to have sex with him? 5. if she burns the food? | Acceptable deemed as responded yes to 1 or more of the 5 questions:   1. No, not acceptable 2. Yes, acceptable 3. Missing |
| Ever experienced any controlling behaviour from partner? | Please tell me if these apply  to your relationship with your (last) husband/partner?   1. He is/was jealous or angry if you talk/talked to other men? 2. He frequently accuses/accused you of being unfaithful? 3. He does/did not permit you to meet your female friends? 4. He tries/tried to limit your contact with your family? 5. He insists/insisted on knowing where you are/were at all times? | Ever experienced any controlling behaviour deemed as responded yes to 1 or more of the 5 questions:   1. No 2. Yes 3. Missing |
| Marital status | Are you currently married or living together with a man as if married? Have you ever been married or lived together with a man as if married? What is your marital status now: are you widowed, divorced, or separated? | 1. Never in union 2. Married 3. Living with partner 4. Widowed 5. Divorced 6. No longer living together/ separated |
| Partner has 1 or more other wives | Does your (husband/partner) have other wives or does he live with other women as if married? | 1. No 2. Yes 3. Missing |
| **CHARACTERISTICS OF HOUSEHOLDS** | | |
| Urban or rural area | Type of place of residence where the respondent was interviewed, created based on whether the cluster or sample point number is defined as urban or rural. | 1. Urban 2. Rural |
| Wealth quintile | Generated by dividing the wealth index score (a continuous variable) into quintiles. The wealth index score is calculated using principal components analysis and is based on the number and types of consumer goods owned by the household, and the types of water access and sanitation facilities present. It is specific to urban and rural areas. | 1. Poorest 2. Poor 3. Middle 4. Richer 5. Richest |
| **CHARACTERISTICS OF PARTNERS** | | |
| Age | How old were you at your last birthday? | 1. 15-29 2. 30-44 3. 45+ |
| Highest level of education | Have you ever attended school? What is the highest level of school you attended:  primary, secondary, or higher? | 1. No education 2. Primary education 3. Secondary education 4. Higher education |
| Currently working | Have you done any work in the last 7 days? | 1. No 2. Yes 3. Missing |
| Drinks alcohol | Question asked of mothers: Does your husband/partner drink alcohol? | 1. No 2. Yes 3. Missing |
